# Supplementary material for: Brain functional-structural gradient coupling reflects development, behavior and genetic influences
Source: Nat Commun. 2026 Apr 9;17:4850. doi: 10.1038/s41467-026-71719-y (PMC13222887; doi:10.1038/s41467-026-71719-y)
Supplement: Supplementary file 1 — Supplementary Information [file 41467_2026_71719_MOESM1_ESM.pdf]

Supplementary Material for "Brain Functional-Structural Gradient Coupling Reflects Development, Behavior and Genetic Influences"

Simiao Gao[1,†], Zhiling Gu[1,†], Shengxian Ding[1,†], Gefei Wang[1], Zhengwu Zhang[2], Hongyu Zhao[1] & Yize Zhao[1,*]

[1] Department of Biostatistics, Yale University, New Haven, CT 06510, USA
[2] Department of Statistics and Operations Research, University of North Carolina at Chapel Hill, Chapel Hill, NC 27599, USA
[†] These authors contributed equally to this work
[*] Correspondence: yize.zhao@yale.edu

Supplementary Tables

Supplementary Table 1. Statistical comparison of heritability between SF gradient coupling and unimodal gradients across cohorts, atlases, and gradient dimensions. For each trait (FCG or SCG), dimension (Global or PC1–PC3), and atlas (D-K or Yeo-7), we report paired-sample t-statistics and two-tailed p-values after the Bonferroni correction comparing SF gradient coupling with the corresponding unimodal gradient in children and adults.

			Children		Adults	
Phenotype	Metric	Subnetwork	t-statistics	adjusted p-value	t-statistics	adjusted p-value
FC Gradient	Global	D-K	40.86	5.78E-240	43.76	2.28E-263
	First Gradient	D-K	6.77	2.77E-08	4.86	5.58E-05
	Second Gradient	D-K	5.06	2.67E-05	8.09	1.08E-10
	Third Gradient	D-K	9.68	1.38E-13	9.49	3.08E-13
	Global	Yeo-7	25.98	8.32E-80	27.75	3.85E-86
	First Gradient	Yeo-7	3.67	1.83E-02	6.90	4.03E-05
	Second Gradient	Yeo-7	2.87	9.42E-02	4.42	3.97E-03
	Third Gradient	Yeo-7	6.41	9.41E-05	5.26	7.64E-04
SC Gradient	Global	D-K	34.30	9.82E-187	-13.58	1.27E-38
	First Gradient	D-K	4.23	5.62E-04	-8.68	9.27E-12
	Second Gradient	D-K	6.81	2.30E-08	-7.66	6.70E-10
	Third Gradient	D-K	5.62	2.96E-06	-10.60	3.22E-15
	Global	Yeo-7	20.90	8.05E-61	-2.45	1.19E-01
	First Gradient	Yeo-7	3.63	1.99E-02	-2.78	1.12E-01
	Second Gradient	Yeo-7	5.10	1.05E-03	-2.43	2.26E-01
	Third Gradient	Yeo-7	5.52	4.68E-04	-3.70	1.71E-02

Supplementary Table 2: Cognitive and mental health measures and their corresponding variable codes in the ABCD and HCP-YA studies. The table lists, for each outcome, the domain, the label used in this manuscript, the dataset-specific variable name, and a brief description of the measure assessed.

Domain	Outcomes	ABCD Codes	HCP-YA Codes	Outcome Dictionary
Mental Health	Internal	cbcl_scr_syn_internal_t	ASR_Intn_T	Internalizing Problems
	External	cbcl_scr_syn_external_t	ASR_Extn_T	Externalizing Problems
	Anx/Dep	cbcl_scr_syn_anxdep_t	ASR_Anxd_T	Anxious/Depressed Problems
	With/Dep	cbcl_scr_syn_withdep_t	ASR_Witd_T	Withdrawn/Depressed Problems
	RuleBreak	cbcl_scr_syn_rulebreak_t	ASR_Rule_T	Rule-Breaking Behavior
	Aggressive	cbcl_scr_syn_aggressive_t	ASR_Aggr_T	Aggressive Behavior
Cognition	PicVocab	nihtbx_picvocab_agecorrected	PicVocab_AgeAdj	Picture Vocabulary
	Flanker	nihtbx_flanker_agecorrected	Flanker_AgeAdj	Flanker Inhibitory Control and Attention
	ListSort	nihtbx_list_agecorrected	ListSort_AgeAdj	List Sorting Working Memory
	CardSort	nihtbx_cardsort_agecorrected	CardSort_AgeAdj	Dimensional Change Card Sort
	ProcSpeed	nihtbx_pattern_agecorrected	ProcSpeed_AgeAdj	Pattern Comparison
	PicSeq	nihtbx_picture_agecorrected	PicSeq_AgeAdj	Picture Sequence Memory
	ReadEng	nihtbx_reading_agecorrected	ReadEng_AgeAdj	Oral Reading Recognition
	CogFluid	nihtbx_fluidcomp_agecorrected	CogFluidComp_AgeAdj	Fluid Composite
	CogCrystal	nihtbx_cryst_agecorrected	CogCrystalComp_AgeAdj	Crystallized Composite
	CogTotal	nihtbx_totalcomp_agecorrected	CogTotalComp_AgeAdj	Total Cognition Composite

Supplementary Table 3: Children vs. adults: overall and sex-stratified sample characteristics. The table reports basic demographics and baseline cognitive and mental health measures used in this manuscript. For continuous variables, values are presented as mean (SD). For sex, sample size and proportion are reported.

Measure	Children (Overall)	Adults (Overall)	Children (F)	Children (M)	Adults (F)	Adults (M)
Age	9.97 (0.63)	28.62 (3.70)	9.95 (0.62)	10.01 (0.63)	29.39 (3.59)	27.73 (3.63)
PicVocab	108.62 (16.95)	109.65 (14.78)	108.10 (16.98)	109.18 (16.90)	107.55 (14.36)	112.04 (14.91)
Flanker	96.27 (13.15)	101.94 (10.00)	95.91 (12.94)	96.66 (13.35)	100.90 (9.72)	103.12 (10.20)
ListSort	101.84 (14.30)	103.55 (13.09)	101.37 (14.23)	102.35 (14.36)	102.83 (13.34)	104.38 (12.78)
CardSort	98.03 (15.14)	102.74 (9.75)	98.52 (14.93)	97.51 (15.35)	102.35 (9.93)	103.18 (9.53)
ProcSpeed	95.69 (21.35)	103.97 (19.65)	96.69 (21.12)	94.61 (21.56)	103.97 (19.08)	103.98 (20.30)
PicSeq	102.46 (16.16)	105.70 (16.39)	103.15 (15.92)	101.71 (16.38)	108.25 (15.51)	102.80 (16.89)
ReadEng	104.08 (19.37)	107.36 (14.76)	103.58 (19.46)	104.62 (19.26)	105.75 (14.52)	109.19 (14.84)
CogFluid	97.79 (16.75)	106.29 (16.81)	98.23 (16.60)	97.30 (16.90)	106.28 (16.70)	106.29 (16.95)
CogCrystal	107.54 (18.35)	110.57 (16.76)	106.97 (18.34)	108.16 (18.33)	108.27 (16.38)	113.18 (16.82)
CogTotal	102.92 (17.42)	114.27 (19.85)	102.85 (17.44)	103.00 (17.40)	112.50 (19.92)	116.29 (19.59)
Anx/Dep	53.40 (5.84)	53.90 (6.33)	53.19 (5.74)	53.62 (5.95)	53.63 (6.17)	54.21 (6.50)
With/Dep	53.22 (5.57)	53.68 (5.85)	52.62 (4.99)	53.87 (6.06)	52.93 (5.40)	54.55 (6.22)
RuleBreak	52.40 (4.47)	53.72 (5.04)	52.26 (4.18)	52.54 (4.76)	53.09 (4.46)	54.44 (5.55)
Aggressive	52.45 (5.02)	52.46 (3.98)	52.12 (4.52)	52.81 (5.49)	52.18 (3.85)	52.79 (4.11)
Internal	48.21 (10.46)	48.40 (10.67)	47.52 (10.45)	48.95 (10.42)	47.89 (10.25)	48.99 (11.12)
External	44.98 (9.92)	48.37 (8.75)	44.45 (9.51)	45.54 (10.31)	46.96 (8.87)	49.99 (8.34)
Sex: Female (F)	2774 (51.9%)	466 (53.3%)				
Sex: Male (M)	2569 (48.1%)	409 (46.7%)				

Supplementary Table 4: Empirical p-values for cell-type enrichment analyses after correction for spatial auto-correlation. This table reports the two-sided nominal p-values for the enrichment of cell-type–specific gene sets in relation to the regional heritability patterns of structural–functional gradient coupling. To account for spatial auto-correlation, p-values were derived from 10,000 spin-rotation permutations [1], separately for the young adult and child cohorts.

**Cohort**	**cell type**	Astrocyte	Endothelial	Excitatory neuron 1	Excitatory neuron 2	Excitatory neuron 3
	**adjusted p-value**	0.046	0.136	0.163	0.100	0.009
	**cell type**	Excitatory neuron 4	Excitatory neuron 5	Excitatory neuron 6	Excitatory neuron 8	Inhibitory neuron 1
	**adjusted p-value**	0.042	0.022	0.135	0.047	0.055
Young Adults	**cell type**	Inhibitory neuron 2	Inhibitory neuron 3	Inhibitory neuron 4	Inhibitory neuron 6	Inhibitory neuron 7
	**adjusted p-value**	0.223	0.426	0.277	0.112	0.173
	**cell type**	Inhibitory neuron 8	Microglia	OPC	Oligodendrocyte	Pericyte
	**adjusted p-value**	0.153	0.015	0.055	0.171	0.181
	**cell type**	Astrocyte	Endothelial	Excitatory neuron L2-3	Excitatory neuron L3-5	Excitatory neuron L4-5
	**adjusted p-value**	0.108	0.015	0.110	0.102	0.115
Children	**cell type**	Excitatory neuron L4-6	Excitatory neuron L5-6	Inhibitory neuron L1-2	Inhibitory neuron L1-3	Inhibitory neuron L1-4
	**adjusted p-value**	0.137	0.119	0.068	0.071	0.055
	**cell type**	Inhibitory neuron L2-4	Inhibitory neuron L3-6	Microglia	OPC	Oligodendrocyte
	**adjusted p-value**	0.053	0.065	0.0004	0.075	0.165

Supplementary Figures

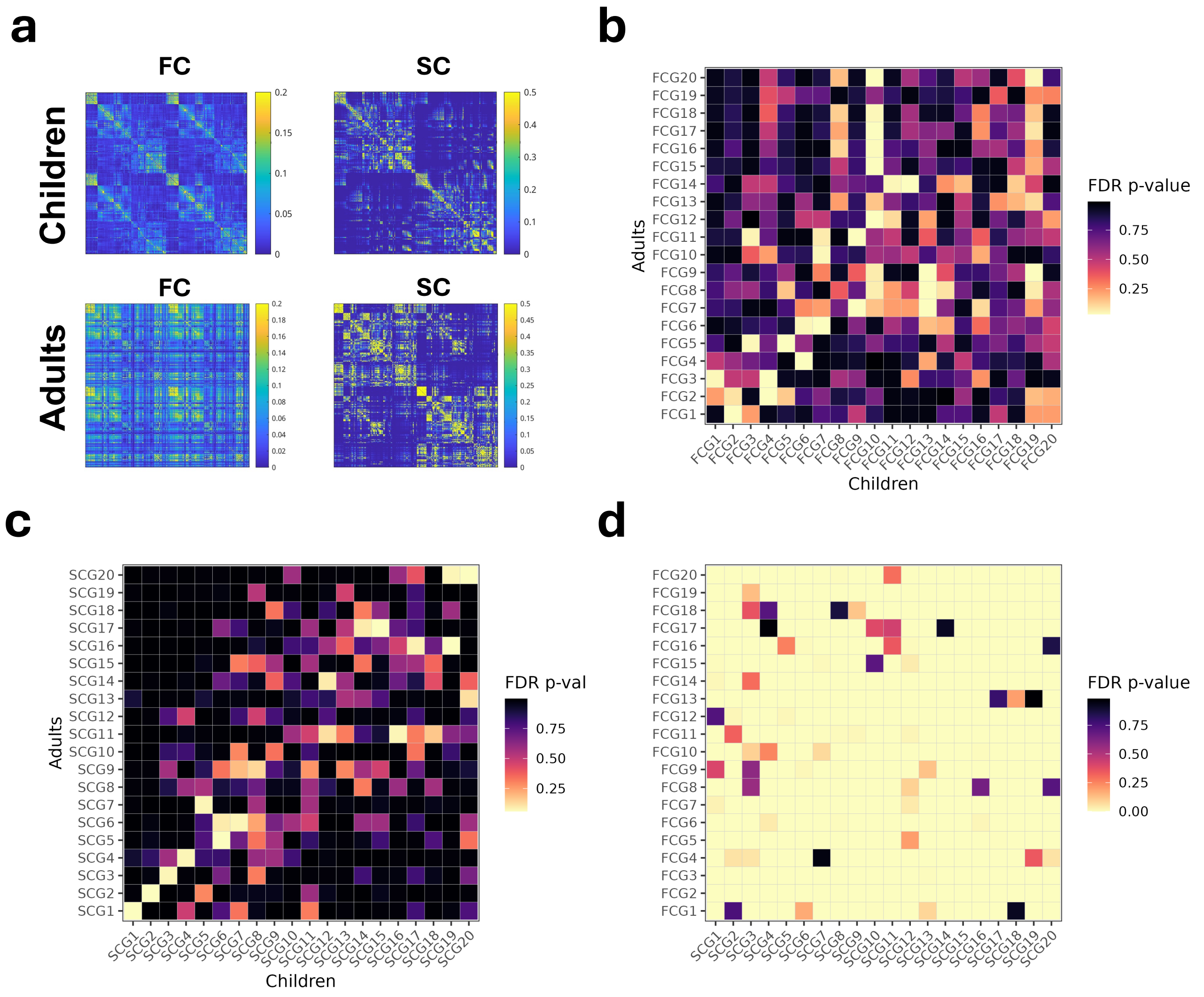

Supplementary Figure 1: **a** Structural and functional connectivity matrices, averaged across subjects in each cohort. **b, c** Heatmaps of FDR-adjusted two-sided p-values from 10,000 times spin-rotation permutation tests comparing children and adults for SCGs and FCGs, respectively. **d** Heatmap of FDR-adjusted two-sided p-values for group differences in SFGCs between children and adults. Source data are provided as a Source Data file.

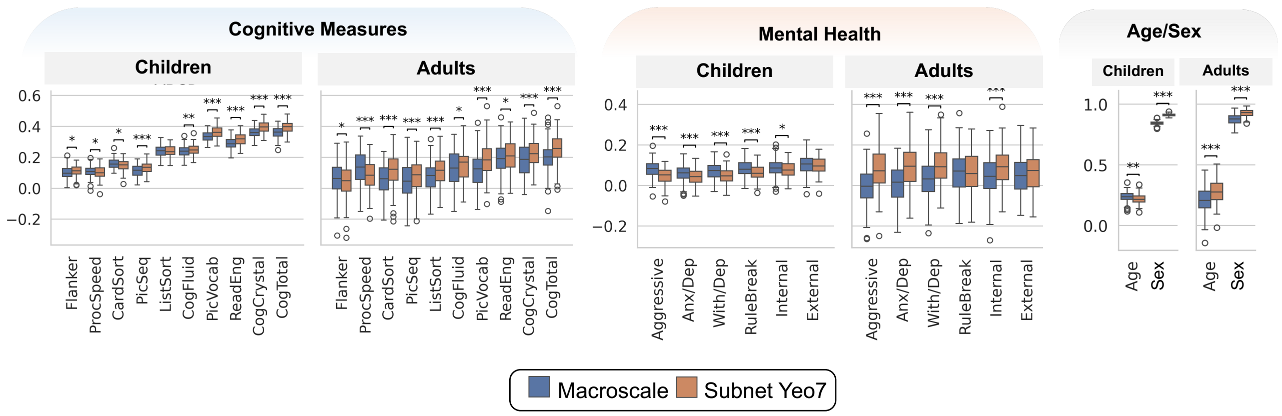

Supplementary Figure 2: Box plots illustrating the association between gradient coupling and behavioral outcomes at the macroscale (blue) and Yeo-7 subnetwork level (orange) using MLP. Correlation coefficients are shown for continuous measures (cognitive and mental health), and AUC values for age and sex. Box plots indicate median (middle line), 25th, 75th percentile (box) and 5th and 95th percentile (whiskers) as well as outliers (single points). Asterisks denote significance level in the difference between macroscale SFGC-outcome associations and subnetwork SFGC-outcome associations (FDR-corrected two-sided paired t-test, $*: p < 0.05$, $**: p < 0.01$, $***: p < 0.001$) using $n = 100$ splitting replicates. Exact p-values are reported in Supplementary Data File. Source data are provided as a Source Data file.

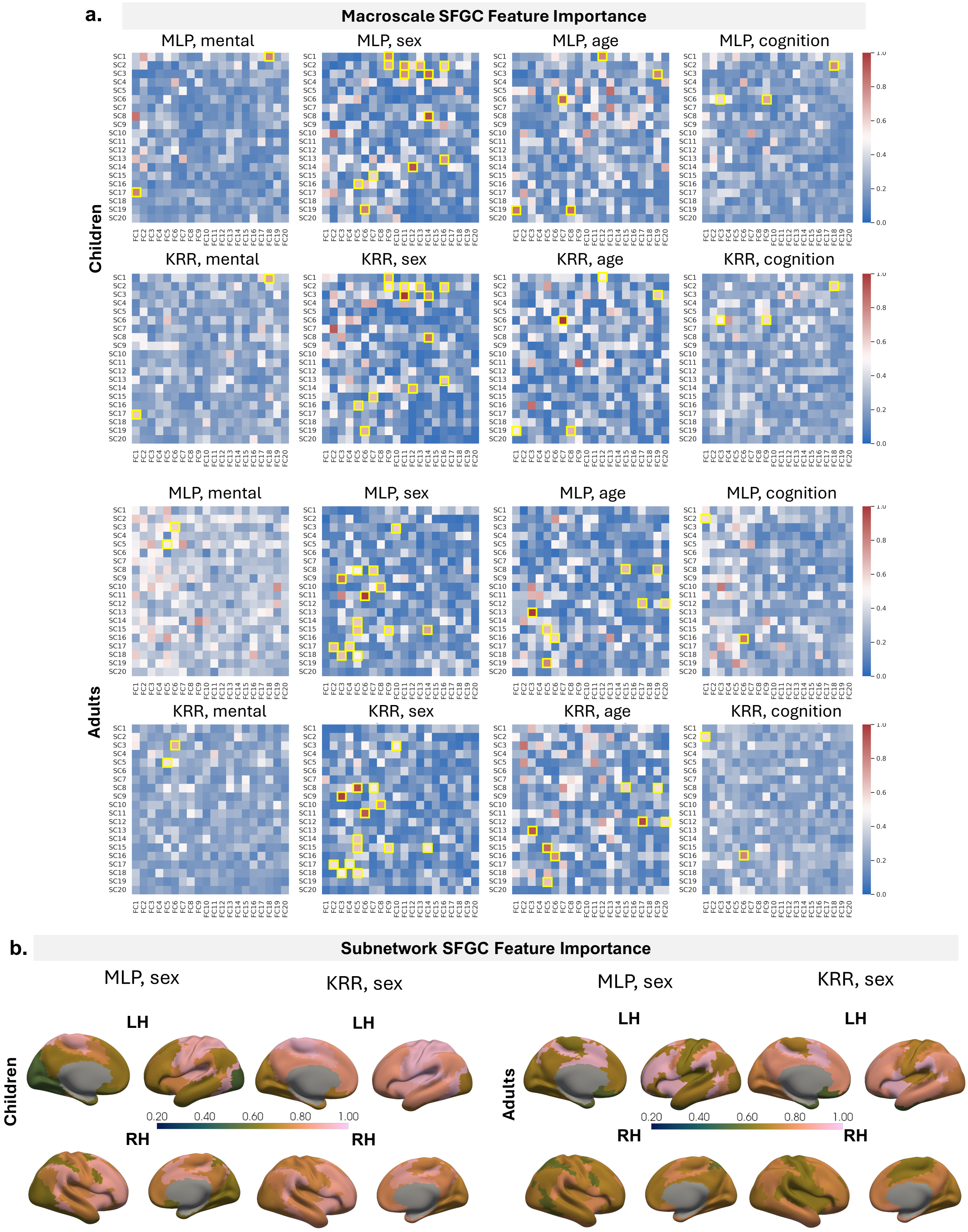

Supplementary Figure 3: Examples of feature importance patterns of the association between SFGC and outcomes using Kernel Ridge Regression (KRR) and multilayer perceptrons (MLP). a Average feature importance of macroscale SFGC's association with mental health outcomes, sex, age, and cognitive outcomes. Key SFGC features with similar patterns between MLP and KRR are highlighted by yellow squares. **b** Feature importance of subnetwork SFGC's association with sex. Colorbars in **a** and **b** indicate the average feature importance. Source data are provided as a Source Data file.

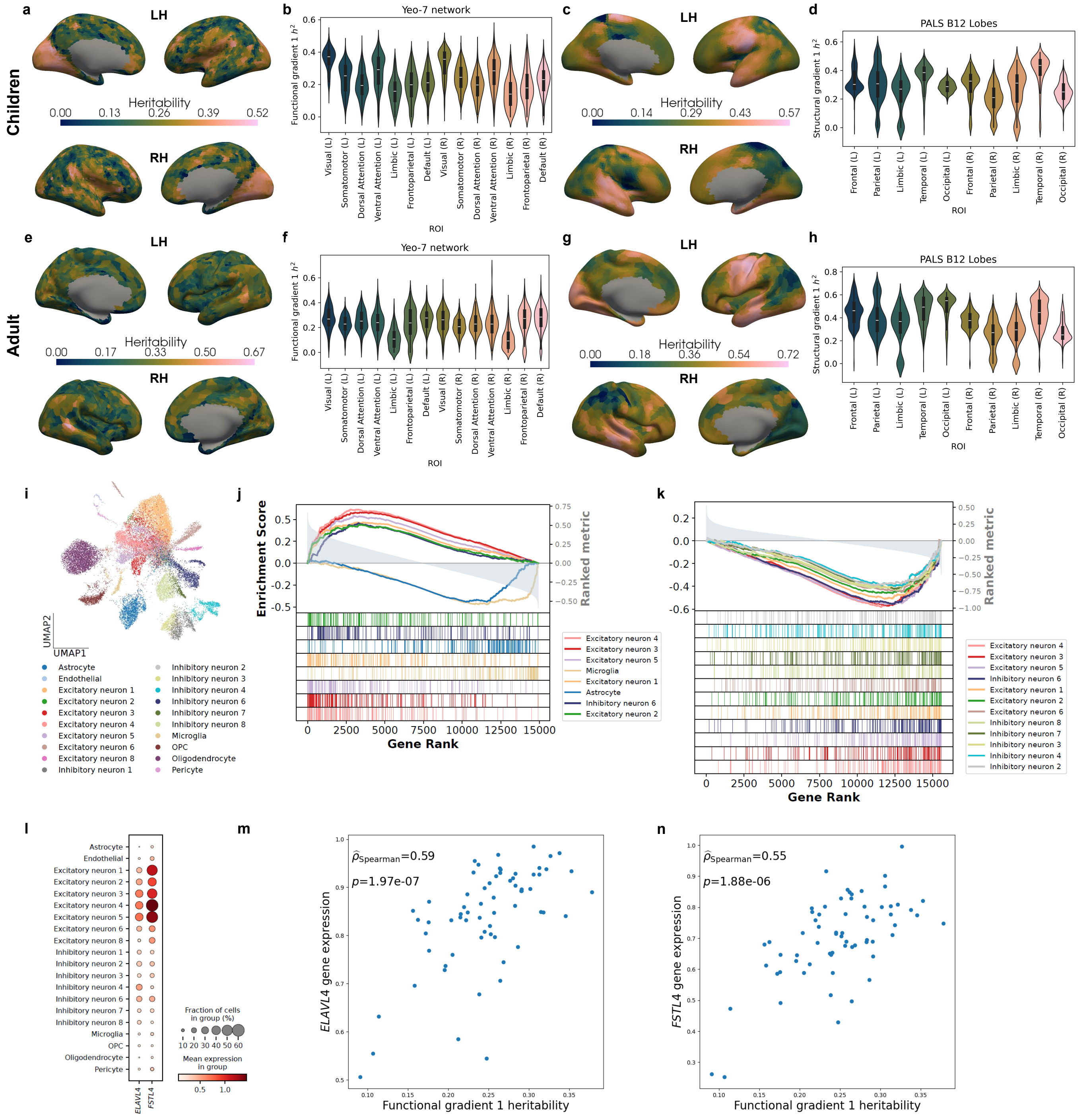

Supplementary Figure 4: Heritability & imaging transcriptomics analyses of functional and structural gradients. **a, e**. Heritability estimates of the first functional gradient for the children and young adult cohorts. **b, f**. Heritability estimates of the first functional gradient grouped by Yeo-7 networks. **c, g**. Heritability estimates of the first structural gradient. **d, h**. Heritability estimates of the first structural gradient grouped by PALS B12 lobes. **i**. Two-dimensional visualization of the single-cell RNA-sequencing dataset reference [2] using UMAP [3]. Cells are colored by their cell type labels. **j, k**. GSEA results of the first functional & structural gradient heritability for the yound adult cohort. Only cell types with absolute values of normalized enrichment scores greater than 2 are shown. **l**. Dot plot of genes *ELAVL4* and *FSTL4*. **m, n**. Scatter plots showing the relationship between the first functional gradient heritability and gene expression values of *ELAVL4* (**m**) and *FSTL4* (**n**). Source data are provided as a Source Data file.

Data Harmonization Details

Supplementary Fig. 5 presents the impact of data harmonization on macroscale SFGC. The post-harmonization plots of the first two principal components exhibit significantly improved randomization across motion levels, sites, and scanners, highlighting the effectiveness of the proposed harmonization strategy, especially in mitigating pronounced motion and scanner effects in the ABCD cohort.

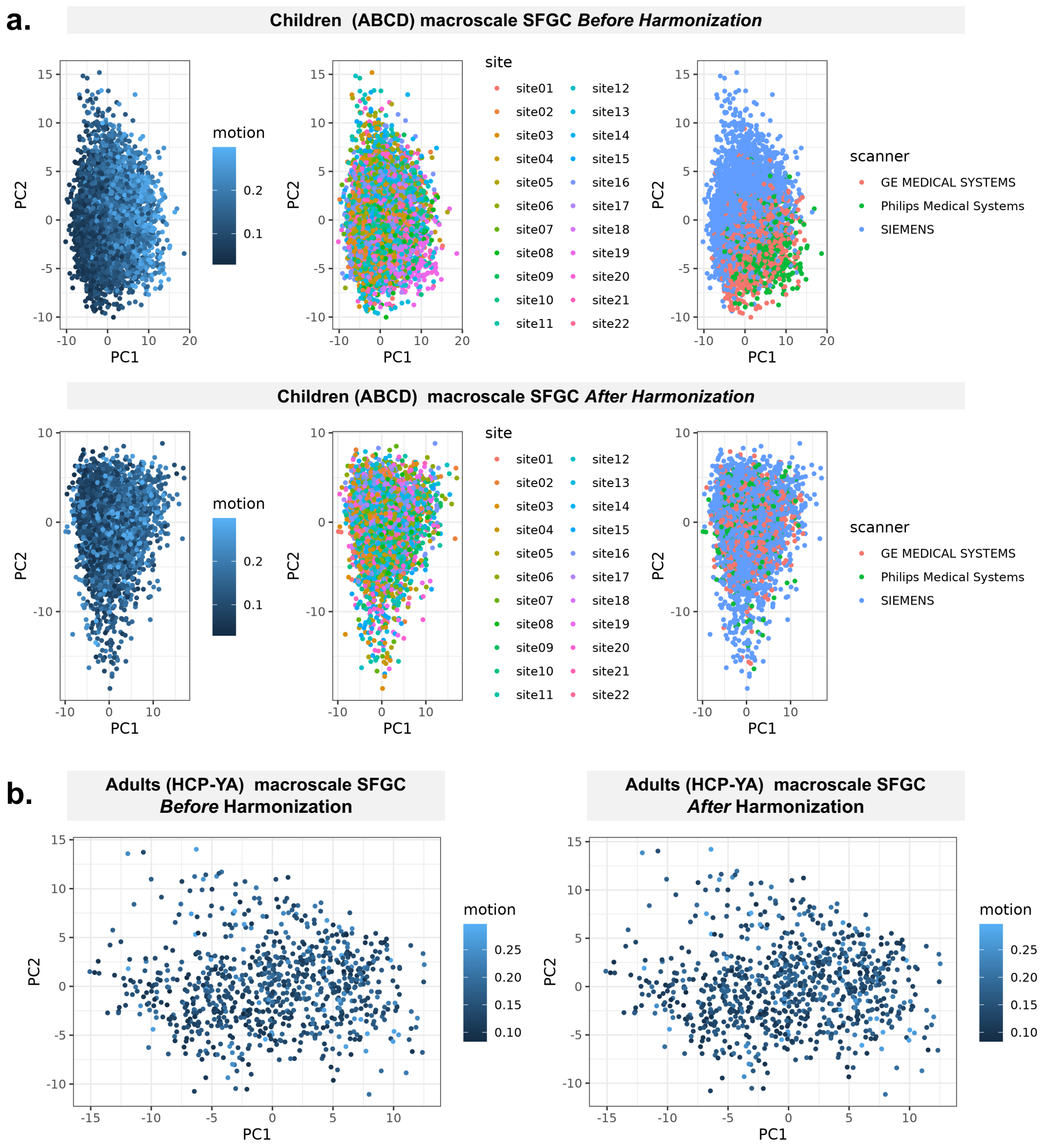

Supplementary Figure 5: The principal components plot of macroscale SFGC before and after data harmonization. a. Children's macroscale SFGC before and after data harmonization colored by motion (mean_FD), sites and scanners. **b.** Adults' macroscale SFGC before and after data hamonization colored by motion. Source data are provided as a Source Data file.

H

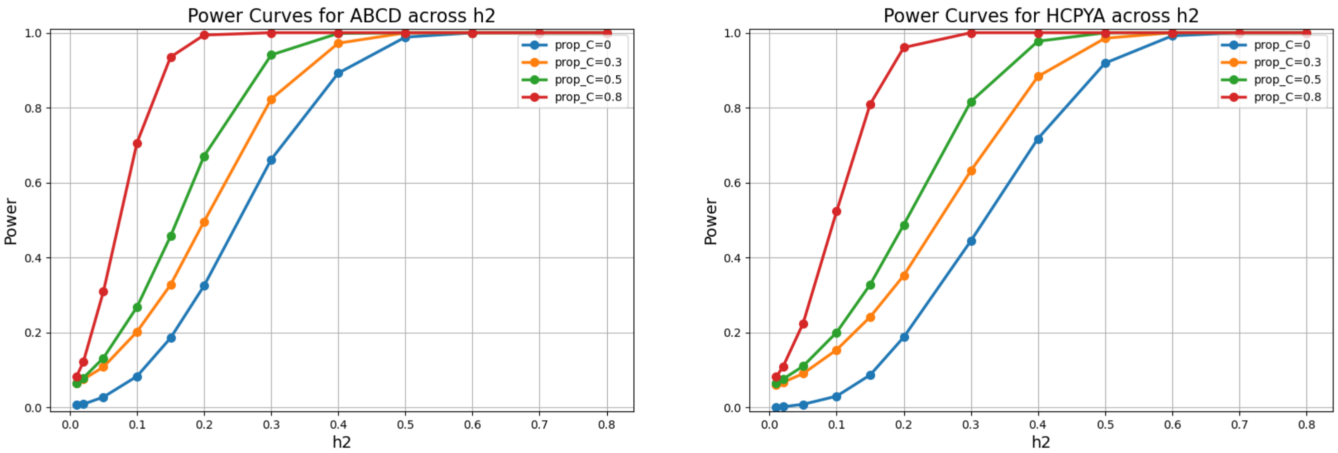

Supplementary Figure 6: Power analyses for detecting heritability. Power curves show the ability to detect nonzero heritability (h^2) under varying proportions of shared environmental variance (prop_C $= \frac{\sigma_C^2}{\sigma_C^2+\sigma_E^2}$). Power was estimated from 5,000 simulations per parameter setting, using each cohort's observed genetic and environmental relatedness matrices. Each panel plots power (y-axis) as a function of the true heritability (x-axis), with separate curves for different prop_C values for the children cohort (left panel) and the young adult cohort (right panel). Source data are provided as a Source Data file.

References

[1] Váša, F. et al. Adolescent tuning of association cortex in human structural brain networks. Cerebral Cortex **28**, 281–294 (2018).

[2] Lake, B. B. et al. Integrative single-cell analysis of transcriptional and epigenetic states in the human adult brain. Nature Biotechnology **36**, 70–80 (2018).

[3] McInnes, L., Healy, J., Saul, N. & Grossberger, L. UMAP: uniform manifold approximation and projection. The Journal of Open Source Software **3**, 861 (2018).
