# Supplementary material for: Brain functional-structural gradient coupling reflects development, behavior and genetic influences
Source: Nat Commun. 2026 Apr 9;17:4850. doi: 10.1038/s41467-026-71719-y (PMC13222887; doi:10.1038/s41467-026-71719-y)
Supplement: Supplementary file 2 — Description of Additional Supplementary Files [file 41467_2026_71719_MOESM2_ESM.pdf]

Description of Additional Supplementary Files

File name: Supplementary Data

Description: This file contains the exact p-values shown in Fig. 3a-c, Supplementary Fig. 2.
