# Supplementary material for: Brain functional-structural gradient coupling reflects development, behavior and genetic influences
Source: Nat Commun. 2026 Apr 9;17:4850. doi: 10.1038/s41467-026-71719-y (PMC13222887; doi:10.1038/s41467-026-71719-y)
Supplement: Supplementary file 4 — Reporting Summary [file 41467_2026_71719_MOESM4_ESM.pdf]

nature portfolio

Corresponding author(s): Yize Zhao

Last updated by author(s): Mar 9, 2026

Reporting Summary

Nature Portfolio wishes to improve the reproducibility of the work that we publish. This form provides structure for consistency and transparency in reporting. For further information on Nature Portfolio policies, see our Editorial Policies and the Editorial Policy Checklist.

Statistics

For all statistical analyses, confirm that the following items are present in the figure legend, table legend, main text, or Methods section.

n/a	Confirmed	
☐	☒	The exact sample size (*n*) for each experimental group/condition, given as a discrete number and unit of measurement
☐	☒	A statement on whether measurements were taken from distinct samples or whether the same sample was measured repeatedly
☐	☒	The statistical test(s) used AND whether they are one- or two-sided<br>*Only common tests should be described solely by name; describe more complex techniques in the Methods section.*
☐	☒	A description of all covariates tested
☐	☒	A description of any assumptions or corrections, such as tests of normality and adjustment for multiple comparisons
☐	☒	A full description of the statistical parameters including central tendency (e.g. means) or other basic estimates (e.g. regression coefficient) AND variation (e.g. standard deviation) or associated estimates of uncertainty (e.g. confidence intervals)
☐	☒	For null hypothesis testing, the test statistic (e.g. *F*, *t*, *r*) with confidence intervals, effect sizes, degrees of freedom and *P* value noted<br>*Give P values as exact values whenever suitable.*
☒	☐	For Bayesian analysis, information on the choice of priors and Markov chain Monte Carlo settings
☒	☐	For hierarchical and complex designs, identification of the appropriate level for tests and full reporting of outcomes
☐	☒	Estimates of effect sizes (e.g. Cohen's *d*, Pearson's *r*), indicating how they were calculated

Our web collection on statistics for biologists contains articles on many of the points above.

Software and code

Policy information about availability of computer code

Data collection	No new data were collected for this study. Data from ABCD, HCP-YA and HCP-D can be obtained as described in the Data Availability Section.
Data analysis	Python 2.7 and 3.12 was used for data processing and analysis. The code used to generate structural and functional gradients, compute gradient coupling, perform association analyses, conduct heritability estimation, and carry out transcriptomic analyses is publicly available on GitHub at: https://github.com/Zhao-team/SF-Gradient-Coupling.<br><br>The dependencies for connectivity matrix construction using SBCI pipeline include:<br>Qt library, version 5.9.5.<br>GNU Compiler Collection (GCC), version 6.5.0.<br>MRtrix3 software for magnetic resonance image processing, version 3.0.4.<br>FreeSurfer software for cortical reconstruction and MRI processing, version 6.0.0.<br>ANTs (Advanced Normalization Tools) for neuroimaging analysis, version 2.3.1.<br>fsl, version 5.0.9.<br>Java, version 10.0.2.<br>MATLAB, version 2019b.<br>dcm2niix software for DICOM to NIfTI conversion, version 1.0.20190902.<br>pigz, a parallel implementation of gzip for modern multi-processor, multi-core machines, version 2.8.<br>Anaconda distribution, which includes Python and a variety of scientific packages, version 4.3.0.<br><br>For packages used for analysis, please refer to the GitHub repository.

For manuscripts utilizing custom algorithms or software that are central to the research but not yet described in published literature, software must be made available to editors and reviewers. We strongly encourage code deposition in a community repository (e.g. GitHub). See the Nature Portfolio guidelines for submitting code & software for further information.

Data

Policy information about availability of data

All manuscripts must include a data availability statement. This statement should provide the following information, where applicable:

- Accession codes, unique identifiers, or web links for publicly available datasets
- A description of any restrictions on data availability
- For clinical datasets or third party data, please ensure that the statement adheres to our policy

Neuroimaging and behavioral data from the Adolescent Brain Cognitive Development (ABCD) Study and the Human Connectome Project Development (HCP-D) cohort are available through the NIH Data Archive (https://nda.nih.gov), subject to data use agreements and approval. Neuroimaging data and most behavioral measures from the Human Connectome Project Young Adult (HCP-YA) cohort are publicly available at https://db.humanconnectome.org, with access to restricted data subject to approval.

Research involving human participants, their data, or biological material

Policy information about studies with human participants or human data. See also policy information about sex, gender (identity/presentation), and sexual orientation and race, ethnicity and racism.

Reporting on sex and gender	Sex (assigned at birth) was included in our model as a binary demographic outcome for both ABCD and HCP datasets.
Reporting on race, ethnicity, or other socially relevant groupings	No socially constructed or socially relevant variables were used in this study.
Population characteristics	ABCD: the final analytic sample consisted of 5,343 subjects (mean age = 9.97 years; 48.1% male). For the heritability analyses, we restricted the sample to 975 subjects drawn from 483 families contributing related pairs, which includes 120 monozygotic (MZ) twin pairs, 176 dizygotic (DZ) twin pairs, and 223 full sibling pairs.<br><br>HCP-YA: Our final analytic sample comprised 875 participants (mean age = 28.62 years; 46.74% male) from from 407 families, including 104 MZ twin pairs, 54 DZ twin pairs, 478 full sibling pairs, 25 half sibling pairs, and 106 singletons.<br><br>The HCP-D dataset was used solely as an intermediate reference for data harmonization and was not included in any downstream analyses. The subset of HCP-D used in the analysis included 93 subjects (mean age = 10.01 years; 38.7% male).
Recruitment	Participants for the ABCD Study were recruited from 21 U.S. research sites using probability sampling of public and private schools, designed to reflect the demographic diversity of the U.S. population aged 9–10 years.<br><br>Participants in the HCP-YA Study were recruited from the Missouri Family Registry and surrounding areas, comprising a sample of healthy adult twins and their non-twin siblings aged 22 to 35 years.<br><br>Participants in the HCP-D Study were recruited across multiple sites in the United States, comprising a population-based sample of subjects aged 5 to 21 years.
Ethics oversight	The ABCD study was approved by the institutional review board of the University of California, San Diego (IRB# 160091). The HCP study was approved by the Washington University Institutional Review Board.

Note that full information on the approval of the study protocol must also be provided in the manuscript.

Field-specific reporting

Please select the one below that is the best fit for your research. If you are not sure, read the appropriate sections before making your selection.

☒ Life sciences ☐ Behavioural & social sciences ☐ Ecological, evolutionary & environmental sciences

For a reference copy of the document with all sections, see nature.com/documents/nr-reporting-summary-flat.pdf

Life sciences study design

All studies must disclose on these points even when the disclosure is negative.

Sample size	Multi-modal imaging and behavioral data were obtained for both ABCD and HCP. The final sample size is determined by the availability of both imaging and cognitive and mental health outcomes. The final sample sizes for ABCD, HCP-YA and HCP-D are 5,343, 875 and 93, respectively.
Data exclusions	For ABCD and HCP-YA:

Subjects with missing outcome measures or incomplete imaging data were excluded.
Subjects with mean framewise displacement greater than 0.3mm were excluded.

For HCP-D:
Subjects with incomplete imaging data were excluded.
Subjects with mean framewise displacement greater than 0.3mm were excluded.
Subjects not aged 9-10 were excluded.

Replication

The models were applied to both the ABCD and HCP datasets. To ensure robustness, we performed 100 random splits of training and testing sets. Standard errors were computed, and two-sample t-tests were conducted to evaluate the statistical validity and reproducibility of the results.

Randomization

The original studies (ABCD and HCP) did not involve experimental randomization, as participants were observationally enrolled based on age and other demographic factors, with no intervention groups. In our analysis, randomization was introduced through 100 random splits of the data into training and testing sets to ensure robustness and generalizability of the predictive models.

Blinding

As the ABCD and HCP datasets involve no group allocation or experimental intervention, blinding procedures were not applicable.

Reporting for specific materials, systems and methods

We require information from authors about some types of materials, experimental systems and methods used in many studies. Here, indicate whether each material, system or method listed is relevant to your study. If you are not sure if a list item applies to your research, read the appropriate section before selecting a response.

Materials & experimental systems

n/a	Involved in the study	
☒	☐	Antibodies
☒	☐	Eukaryotic cell lines
☒	☐	Palaeontology and archaeology
☒	☐	Animals and other organisms
☒	☐	Clinical data
☒	☐	Dual use research of concern
☒	☐	Plants

Methods

n/a	Involved in the study	
☒	☐	ChIP-seq
☒	☐	Flow cytometry
☐	☒	MRI-based neuroimaging

Plants

Seed stocks

N/A

Novel plant genotypes

N/A

Authentication

N/A

Magnetic resonance imaging

Experimental design

Design type

Resting state

Design specifications

The ABCD scan session followed a fixed sequence, beginning with a brief localizer for head alignment, followed by the acquisition of high-resolution 3D T1-weighted structural images, two runs of eyes-open resting-state fMRI, diffusion-weighted imaging, and 3D T2-weighted structural scans. Depending on real-time motion monitoring, one or two additional resting-state fMRI runs were acquired to optimize data quality. More details can be found here: https://www.sciencedirect.com/science/article/pii/S1878929317301214.

The HCP protocol was distributed across four approximately 1-hour sessions over two days, each beginning with a rapid localizer to ensure precise head positioning. Day 1 combined ultra-high-resolution structural imaging (T1- and T2-weighted) with alternating blocks of resting-state and task-evoked fMRI. Day 2 emphasized multi-shell diffusion acquisitions and included additional resting-state and task fMRI runs to enhance test–retest reliability. More details can be found here: https://www.humanconnectome.org/hcp-protocols-ya-3t-imaging#:~:text=Structural%2C%20fMRI%2C%

20and%20dMRI%20acquisitions%20were%20collected,sessions%2C%20each%20approximately%201%20hour%20in%20duration.

Behavioral performance measures

No behavioral performance was assessed, as the study did not involve any task-based paradigms or analyses.

Acquisition

Imaging type(s)

T1-weighted and diffusion MRI images, resting-state fMRI.

Field strength

3T

Sequence & imaging parameters

The ABCD Study used gradient-echo EPI sequences for functional MRI and spin-echo EPI for diffusion MRI. All scans were acquired using a standardized echo planar imaging (EPI) protocol across sites. Functional and diffusion images had a field of view of approximately 216 × 216 mm, with a matrix size of 108 × 108 and 2.4 mm isotropic slice thickness in axial orientation. The functional imaging parameters included a TR of 800 ms, TE of 30 ms, and flip angle of 52°. More details can be found here: https://www.sciencedirect.com/science/article/pii/S1878929317301214.

The HCP-YA cohort employed gradient-echo multiband EPI sequences for fMRI and spin-echo EPI for diffusion imaging, using a customized Siemens Connectome Skyra scanner. Functional images had a field of view of 208 × 180 mm, a matrix size of 104 × 90, and 2 mm isotropic slice thickness acquired in oblique axial orientation. The fMRI sequence was configured with a TR of 720 ms, TE of 33.1 ms, and flip angle of 52°. More details can be found here: https://www.humanconnectome.org/hcp-protocols-ya-3t-imaging#:~:text=Structural%2C%20fMRI%2C%20and%20dMRI%20acquisitions%20were%20collected,sessions%2C%20each%20approximately%201%20hour%20in%20duration.

For HCP-D cohort, scanning protocols largely follow the HCP-YA framework, with modifications necessitated by scanner changes, session length constraints, and the practicalities of imaging young and older cohorts. More details can be found here: https://www.sciencedirect.com/science/article/pii/S1053811918318652?via%3Dihub.

Area of acquisition

Whole brain

Diffusion MRI ☒ Used ☐ Not used

Parameters

ABCD: # of directions: 96 total; b-values: 500, 1000, 2000 s/mm²; Shell type: Multi-shell
HCP-YA: # of directions: 270 total; b-values: 1000, 2000, 3000 s/mm²; Shell type: Multi-shell
HCP-D: # of directions: 370; b-values: 1,500 and 3,000 s/mm2; Shell type: Multi-shell

Preprocessing

Preprocessing software

FreeSurfer: v6.0; cortical surface reconstruction via the recon-all pipeline.
FSL/AFNI tools: Used for brain extraction, motion correction, B0 distortion correction, and spatial normalization.
Segmentation: Atlas-free
Smoothing: Surface-based smoothing with a 5mm FWHM Gaussian kernel.

Normalization

Rigid, affine, and nonlinear

Normalization template

MNI152

Noise and artifact removal

Artifact and structured noise removal was performed during resting-state fMRI preprocessing. Motion parameters were estimated using rigid-body realignment to the first frame of each scan. To correct for physiological and tissue-related noise, nuisance regression included six motion parameters, principal components of white matter and cerebrospinal fluid signals, and global signal regression. Surface-based projection was applied after bias-field correction and brain masking.

Volume censoring

ABCD: Any volume (time point) with FD>0.2mm was excluded from further variance and correlation computations.

HCP: No censoring.

Statistical modeling & inference

Model type and settings

We employed kernel ridge regression (KRR) and multilayer perceptron (MLP) models to examine associations between behavioral outcomes and the proposed gradient-based metric. In addition, we conducted heritability and transcriptomic analyses to investigate the genetic underpinnings of gradient coupling.

Effect(s) tested

This study tested the associations and predictive utility of SFGC for cognitive and mental health traits. We also examined the effects of age and sex on SFGC. Furthermore, we tested the heritability of these metrics across developmental stages and evaluated their spatial correspondence with cell-type-specific gene expression profiles.

Specify type of analysis: ☐ Whole brain ☐ ROI-based ☒ Both

Anatomical location(s)

Yeo-7 subnetworks and D-K atlas were employed.

Statistic type for inference
(See Eklund et al. 2016)

Analyses were conducted on on a region-wise basis across the cortical surface.

Correction	Multiple comparison corrections were performed using the False Discovery Rate and Bonferroni procedures.

Models & analysis

n/a	Involved in the study
☐	☒ Functional and/or effective connectivity
☒	☐ Graph analysis
☐	☒ Multivariate modeling or predictive analysis

Functional and/or effective connectivity	We constructed the functional and structural connectivity (FC and SC) using the Surface-Based Connectivity Integration pipeline, a surface-based, atlas-free approach.
Multivariate modeling and predictive analysis	Functional and structural gradients (FCG and SCG) were derived from FC and SC matrices using diffusion map embedding, a nonlinear dimensionality reduction technique. To quantify alignment between structural and functional hierarchies, we computed the cosine similarity between corresponding FCG and SCG components, forming a measure of SFGC. These SFGC values were then used as predictors in two predictive modeling frameworks: KRR and MLP. Behavioral and demographic variables (e.g., cognition, mental health, sex) served as outcomes. In addition, we conducted heritability and transcriptomic analyses to investigate the genetic underpinnings of gradient coupling.
